# Promoting Patient Safety Through Patient Engagement at the Organisational Level: A Delphi‐Based Needs Assessment Among Patient and Family Advisory Councils

**DOI:** 10.1111/hex.70319

**Published:** 2025-06-10

**Authors:** Larissa Brust, Yannick Blum, Matthias Weigl

**Affiliations:** ^1^ Institute for Patient Safety (IfPS), Medical Faculty University Hospital Bonn Bonn Germany

**Keywords:** competence, Delphi technique, health education, healthcare organisation, patient and family advisory councils (PFAC), patient participation, patient safety

## Abstract

**Background:**

Patient and family advisory councils (PFACs) are increasingly recognised as a promising approach for improving patient safety (PS) through patient engagement (PE) at the organisational level. However, PFAC stakeholders often lack the necessary knowledge and competence to engage effectively in PS‐related issues with healthcare organisations. Moreover, evidence on specific needs for knowledge and competence improvement remains limited, hindering the development of future interventions.

**Objective:**

This study aimed (a) to identify needs for PS‐related competency and knowledge improvement among PFAC stakeholders and (b) to assess current and desired levels of PFAC engagement, roles and factors influencing PFACs' work.

**Design:**

We established an exploratory mixed‐methods design with a modified, two‐round Delphi approach. We first used qualitative content analysis to analyse interview data (Round 1) and then consolidated statements for a quantitative questionnaire (Round 2). Responses were analysed descriptively and for consensus (criterion: 85% agreement). Mixed‐methods analysis was conducted sequentially and convergently.

**Setting and Participants:**

PFAC stakeholders are affiliated with German healthcare organisations, including patient representatives and professionals from healthcare organisations.

**Main Variable and Outcome Studied:**

(a) Needs for competency improvement on PS and communication, self‐assessed knowledge and preferred training formats and (b) PFAC engagement levels, roles and factors influencing PFACs' work.

**Results:**

Across 6 different oncology‐focused PFACs from German university hospitals, 19 stakeholders participated across both rounds. Seventeen needs for competency improvement in PS and communication were identified. After establishing consensus, 10 distinct domains of need were agreed upon (e.g., PS fundamentals, legal basis for PE and respectful communication). While PFAC engagement in PS was inconsistent, participants expressed a strong desire for further involvement. Key implementation factors included limited access to organisational processes, lack of resources and unequal conditions between research‐ and care‐oriented councils.

**Discussion and Conclusion:**

This study highlights the need for targeted training and structural support to strengthen PFACs' role in PS. Competency improvement and role clarity were deemed essential for effective collaboration. Enhancing PFAC engagement in PS requires tailored educational programmes, transparent structures and institutional support. This study provides an empirical basis for interventions to improve PE in PS at the organisational level.

**Patient or Public Contribution:**

A patient representative was actively involved throughout the research process, contributing to the development of study materials and providing independent feedback on interview guides and questionnaires. Her input helped to shape the materials, improve their accessibility to lay audiences and ensure the inclusion of patient‐relevant issues. The research team discussed her feedback in detail and revised study materials accordingly. Beyond the content presented in this manuscript, she contributed to shaping a subsequent intervention that emerged from the study's needs assessment, which was designed as a participatory approach to incorporate patient and stakeholder perspectives from the outset. In addition, she and participating stakeholders of the patient advisory councils are committed to disseminating project findings and developing recommendations to help translate research into practice from a patient perspective.

**Clinical Trial Registration:**

The study was pre‐registered in the German Clinical Trials Register (ID: DRKS00034733).

## 1 Introduction

Patient safety (PS) remains a persistent challenge in healthcare systems worldwide, with preventable harm arising from issues such as medication errors, falls and healthcare‐associated infections [1, 2]. It requires comprehensive strategies that go beyond traditional clinical approaches by incorporating involvement and perspectives of all stakeholders, with particular emphasis on patients' contributions [3, 4]. One increasingly recognised approach is patient engagement (PE). PE refers to collaboration between patients, families, their representatives and healthcare providers in care processes at multiple levels: direct care, organisational design and policy‐making [5]. Prior research has demonstrated potential benefits and growing recognition of PE at the organisational level for PS [4, 5, 6, 7, 8]. Successful examples of PE in PS include patient incident reporting systems or hospital redesign initiatives involving patient representatives, which have contributed to reducing falls and medical errors [9, 10]. Beyond those benefits, broader involvement of patients and their representatives in care processes is often conceived as a moral imperative [8].

The WHO's Global Patient Safety Action Plan highlights PE at the organisational level as a key element in improving PS [3]. However, despite increasing awareness, broader and systematic implementation of PE in healthcare organisations remains scarce [11, 12, 13, 14]. Many challenges in the course of PE implementation are still poorly understood, highlighting the need for further research to fully leverage the potential of PE at the organisational level in enhancing PS [11, 12, 15, 16, 17].

One particularly promising PE measure at the organisational level is the patient and family advisory council (PFAC) [9, 12, 15]. PFACs are composed of patient representatives as well as healthcare organisations (i.e., managerial professionals, healthcare providers and clinicians). These forums aim to facilitate collaboration between patients and healthcare organisations [15]. PFACs contribute to quality and safety improvement initiatives by providing feedback on hospital safety practices, co‐designing educational materials and participating in policy development [6, 9]. Recent literature shows that PFACs have been widely implemented in the United States, Canada, the United Kingdom and Australia [9, 18, 19, 20, 21], with initial efforts in Europe as well [9, 21, 22, 23, 24]. Despite their recognised benefits, PFACs, like other PE initiatives at the organisational level in PS, face significant barriers that impede successful implementation in healthcare organisations [15, 21, 25].

Latest investigations have systematically examined implementation factors associated with institutionalising PE [7, 13, 16, 21]. Therein, two key challenges were reported: First, patient representatives and other PFAC stakeholders often lack necessary knowledge and competencies related to PS, PE and communication with institutional members or healthcare professionals [7, 15, 16, 25, 26]. Accordingly, education and training opportunities have been identified as key factors for successful PE, as also highlighted in a recent scoping review [7, 13, 16, 21]. Yet, structured programmes to overcome these challenges are still scarce [27, 28, 29]. Moreover, little systematic research has been conducted on the specific educational needs of patient representatives in PS [30]. This is in contrast to other topics such as evidence‐based medicine, health economics or even specific aspects of communication or general knowledge needs [31, 32, 33]. This shortcoming persists, although previous efforts continuously placed PS among top training priorities [3, 29, 34]. Tailoring interventions to the specific needs of PFAC stakeholders could substantially enhance their role and impact in healthcare settings [32]. Secondly, the literature suggests that PFACs operate in different contexts, with varying and often poorly defined roles, responsibilities and decision‐making authority [21, 22, 31, 35, 36]. These variations, likely shaped by national and cultural differences in health systems, hamper generic insights on successful implementation and translation for healthcare systems that seek to uptake or expand PE approaches in PS, not least due to the lack of clear success criteria and outcome measurements [21, 37, 38]. So far, few country‐ or system‐specific studies have addressed these issues [22, 31], and none have focused on Germany.

In light of the available literature, persistent shortcomings in the knowledge base on PE at the institutional levels of healthcare organisations remain. There is still limited knowledge of the specific educational and training needs of PFAC stakeholders regarding PS and their collaboration with healthcare organisations. Furthermore, evidence‐guided information on roles, responsibilities and both the current and desired levels of PFAC engagement in PS is limited. These gaps resonate well with findings of previous systematic reviews that call for more empirical evidence to support the development and implementation of PFACs [9, 12, 21, 29]. Addressing these gaps is crucial for advancing engagement of PFACs in healthcare organisations and improving both PS and quality of care. To this end, our investigation sought to provide a deeper understanding of the current state and requirements for future development and successful implementation of PE and PS initiatives at the organisational level. Specifically, this study aimed to

a. identify the needs of PFAC stakeholders, particularly in terms of knowledge and competencies related to PS and collaboration with healthcare organisations (primary objective);
b. assess the current and desired level of PFAC engagement and focus on PS, clarify existing and potential roles of PFACs and analyse factors influencing PFACs' work (secondary objective) and thus
c. inform development, design and implementation of future interventions aimed at improving PS knowledge and PFAC engagement in healthcare organisations (third objective).

## 2 Materials and Methods

### 2.1 Study Design

**Figure 1:**
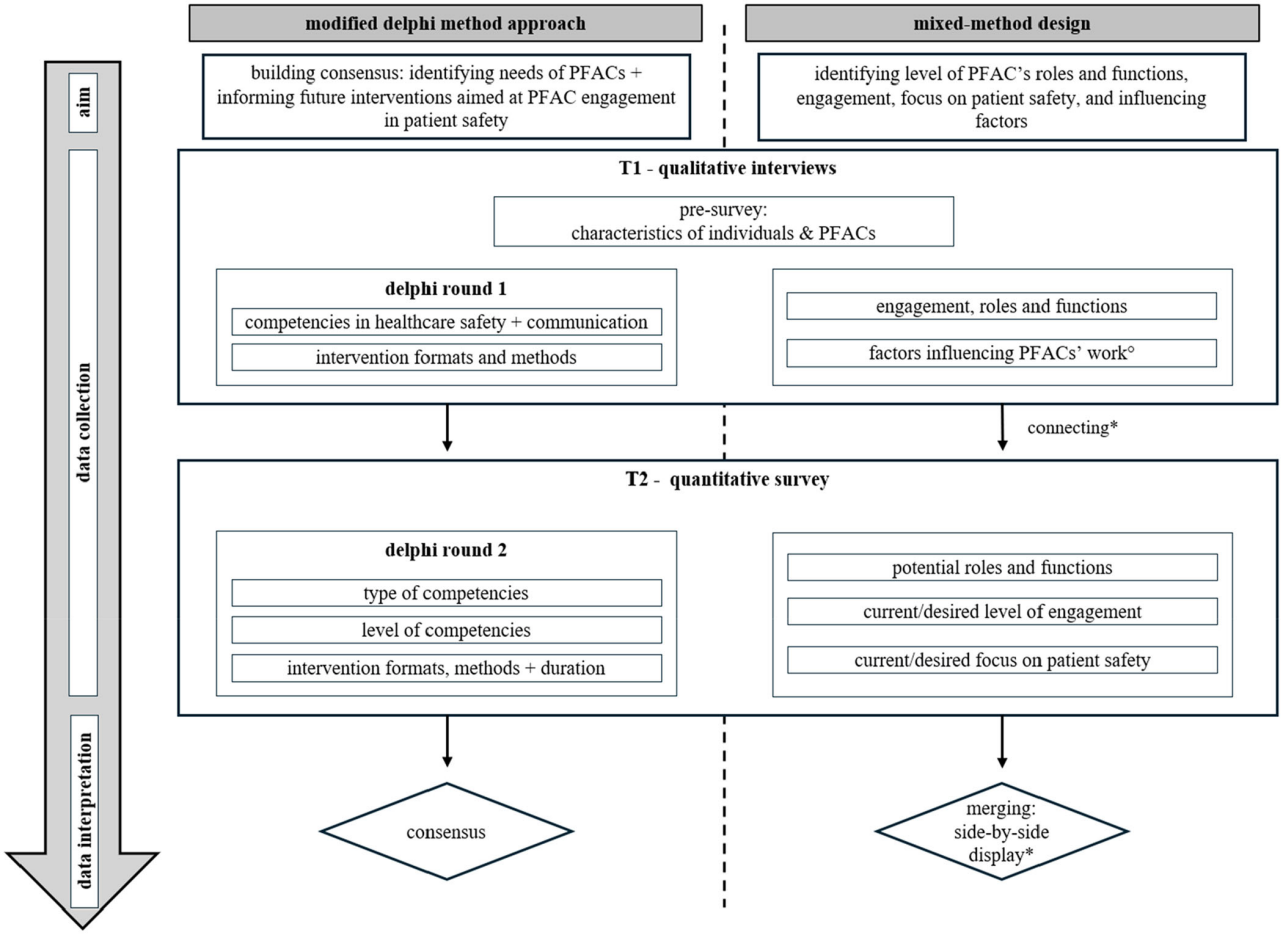
Flow of the study and data integration. *mixed‐methods approaches driven by the motives of development, complementarity and triangulation [40]; °only qualitatively collected.

This study used a sequential and convergent mixed‐methods design with a modified two‐round Delphi approach, consisting of sequentially ordered qualitative and quantitative data assessment parts. Modification involved a predefined number of rounds [39], facilitating a participatory and collaborative consolidation process to efficiently identify the target group's needs. Figure 1 illustrates the study flow, including aims, mixed‐methods motives, data collection rounds and methods of data interpretation.

This study was conducted as part of a larger project on PE in PS (PEPS‐3; acronym for ‘Implementation of patient engagement for the strategic promotion of patient safety’). This article is reported in accordance with the proposal for reporting guidelines on Delphi techniques in health sciences [41] and the Mixed Methods Article Reporting Standards (MMARS) [42].

### 2.2 Participant Selection, Sampling and Recruitment

This study was conducted in various federal states of Germany, focusing on PFACs affiliated with a healthcare organisation. In this study, PFACs refer to a structured group of patients, patient representatives, family members, hospital representatives or healthcare professionals. This definition also includes groups that may not necessarily be explicitly labelled as ‘PFACs’ (e.g., roundtable of patient‐centred organisations).

To identify relevant PFACs and their stakeholders, we employed a purposive and convenience sampling approach. A study team member manually searched for PFACs, collecting contact information, location, affiliation and specialty (e.g., oncology and mucoviscidosis). Since most identified PFACs focused on oncology and were already embedded in institutional settings, we exclusively contacted oncology‐focused PFACs. Invitations were sent via email. Overall, we aimed to recruit at least five PFACs, each represented by 1–3 stakeholders, resulting in a total sample size of at least *n* = 15 participants—an appropriate sample size for an exploratory study in an area that has been little studied [43].

Eligibility criteria included being ≥ 18 years and German‐speaking and having one of the following roles in an oncology‐focused PFAC affiliated with a healthcare organisation for at least 3 months: (a) patient, family member, other caregiver and patient representative; (b) representative of the PFAC‐affiliated healthcare organisation (e.g., quality or risk manager, physician, nurse, directors and administrative staff) or (c) other PFAC stakeholder (e.g., moderator, coordinator and scientist). Detailed information on the professional background and roles of surveyed PFAC members, hospital professionals and characteristics of the participating healthcare organisations is provided in Table 1.

**Table 1:** Characteristics of surveyed participants and PFACs.

Characteristics	
Individual participants	*N* = 19
Age (years), mean ± SD	57.9 ± 12.8
Duration of membership in the PFAC (months), mean ± SD	30.7 ± 29.9
Workload for the PFAC per month (h), *n* (%)	
< 5	7 (36.8)
5–10	8 (42.1)
11–20	1 (5.3)
> 20	3 (15.8)
Role in the PFAC, *n* (%)	
Patient, relative or representative (e.g., from a patient organisation)	16 (84.2)
Clinical representative#	3 (15.8)
Quality and risk manager	1 (5.3)
Moderator	2 (10.5)
Other (i.e., coordinator or psychologist)	2 (10.5)
Motivation for engaging in a PFAC, *n* (%)	
Personal experience as a patient	17 (89.5)
Initiating improvements in the healthcare system	16 (84.2)
Active participation in healthcare decision‐making	12 (63.2)
Networking	9 (47.4)
Personal growth	5 (26.3)
Experience of relatives	4 (21.1)
Professional activity in the PFAC	3 (15.8)
Other	3 (15.8)

Patient and family advisory councils	*N* = 6*
Affiliated with a university hospital (yes), *n* (%)	19 (100)
Regularity of meetings of the PFAC, *n* (%)	
Monthly	4 (21.1)
Quarterly	10 (52.6)
Unregular	5 (26.3)
Number of stakeholders in the PFAC, *n* (%)	
< 5	2 (10.5)
5–10	10 (52.6)
11–15	5 (26.3)
16–20	1 (5.3)
> 20	1 (5.3)
Existing roles in the PFAC (yes), *n* (%)	
Patient, relative or representative (e.g., from a patient organisation)	19 (100)
Clinical representatives	6 (31.6)
Physician	4 (21.1)
Quality and risk manager	3 (15.8)
Moderator	8 (42.1)
Other (e.g., coordinator, self‐help officer, psychologist, scientists, patient care coordinator and consultant associations)	8 (42.1)

Abbreviations: PFAC, patient and family advisory council; SD, standard deviation.

^#^ Participants can have multiple roles.

* Participants were asked about their respective PFAC; therefore, the sample is the same as the individual participants (*n* = 19), and the results are not based on the sample size related to the PFAC.

The study was pre‐registered in the German Clinical Trials Register (ID: DRKS00034733) and was approved by the Ethics Committee of the University of Bonn (Ref. nr: 2024‐236‐BO). Data collection and processing were carried out in accordance with the Declaration of Helsinki, the European Union's General Data Protection Regulation (GDPR) and the German Federal Data Protection Act (BDSG). All participants of the study signed an informed consent, and participants received a monetary compensation of 60€ for their participation.

### 2.3 Researcher Description and Patient Involvement

The research team consisted of two psychologists, one physician and two physiotherapists with experience in qualitative, quantitative and mixed methods in health services, as well as PS and PE research. Their diverse backgrounds helped to shape research design and informed the data collection process. To ensure a patient‐centred perspective, an independent patient representative was actively involved as a collaborator in the study design and development of materials, ensuring their accessibility to lay audiences and encouraging reflexivity within the research team through regular discussions. This participatory approach, combined with the broader needs assessment of PFAC stakeholders, embedded patient and stakeholder perspectives from the outset. In addition, the patient representative and PFAC stakeholders will support the dissemination of findings and the development of recommendations within the larger project, further strengthening the study's proximity to patient priorities [44].

### 2.4 Data Collection

Six key criteria were applied to ensure the quality and reliability of the data collection process and results [44]. The data collection process consisted of two Delphi rounds: (T1) qualitative interviews and (T2) quantitative survey (Figure 1). Data were collected between August 2024 and October 2024. Before the first round, participants were asked to fill in a short pre‐survey (~10 min) where they reported on demographics, roles and workload for the PFAC. Additionally, they were asked about the organisation, structure and roles of their PFAC, as well as the collaboration of the affiliated healthcare organisation and the current level of engagement of the PFAC.

#### 2.4.1 T1—Qualitative Interviews (Round 1)

Qualitative semi‐structured interviews with participants were used to report levels of knowledge and competencies in PS, communication and desired formats and methods for future education interventions. In addition, the current and desired roles and functions of PFACs, their engagement in care and safety issues, and influencing factors were surveyed. Two study team members (L.B. and Y.B.) developed, and two others (M.W. and N.G.) revised the interview guide. After testing for clarity by a patient representative, final adjustments were made. Interviews were based on four topics using open‐ended questions: (a) engagement, roles, and functions of PFACs, (b) knowledge/competencies, (c) factors/conditions influencing PFACs' work and (d) intervention formats and methods (see final guide in Supporting Information 1). Participants were provided with the interview guide and a definition of PS before the interview. Interviews were conducted via video‐communication software application (by L.B. and Y.B.) and audio‐visually recorded and transcribed via ZoomX (Zoom Video Communications Inc., San José, the United States; Deutsche Telekom, Bonn, Germany). All interviews were held individually, with a maximum duration of 40 min. Automatically generated written transcripts (via MAXQDA, VERBI Software GmbH, Berlin, Germany [45]) were reviewed, corrected if necessary and pseudonymised by a study member.

#### 2.4.2 T2—Quantitative Survey (Round 2)

An online questionnaire was used to consolidate the first round's statements and domains and obtain consensus on key features of future interventions (i.e., desired content, duration and competence level). Participants were also asked to assess their level of competencies about PS and communication models and concepts according to Bloom's taxonomy, which should be addressed in an intervention [46]. Additionally, roles and functions, as well as the level of engagement and focus on PS of PFACs, were quantified to further complement and triangulate acquired qualitative data [47]. Survey items were derived from the interview data (Round 1). Additionally, a patient representative assessed lay comprehensibility. Competency domains had to be rated on a 5‐point Likert scale ranging from ‘totally disagree’ to ‘totally agree’. The desired duration for an intervention (i.e., educational module) was assessed in four different predefined time blocks (e.g., < 1 h, 1–2 h, 2–4 h and 4–8 h) and in the category ‘no opinion/unsure’. The current level of engagement was rated according to the common PE levels (i.e., from information to shared leadership) [5]. The perceived current right of participation was rated on a 6‐point Likert scale from 1 ‘very good’ to 6 ‘unsatisfactory’ (according to the German school grading system). At the end, a free text field was provided for additional comments. The final questionnaire is presented in Supporting Information 2. The 10‐min survey was provided via unipark.de (Tivian XI GmbH, Cologne, Germany).

### 2.5 Data Analysis

Qualitative data were analysed using qualitative content analysis with a deductive‐inductive and consensual approach in software MAXQDA [45]. Main categories were predefined based on interview guide topics. Coding rules and definitions of main categories were developed by two authors (L.B. and Y.B.). These authors independently coded randomly selected 30% of the interview transcripts, aiming for an inter‐rater reliability of *K_n_* = 0.80 (kappa coefficient, *K_n_*) [45, 48, 49]. Discrepancies were resolved through discussions, iteratively refining the main category system until *K_n_* = 0.81 was reached. All transcripts were then coded with main categories. Subcategories were inductively and iteratively developed: two team members independently proposed subcategories based on 20% of transcribed material and then synthesised, pilot‐tested and refined via coding of an additional 20%. Upon reaching *K_n_* = 0.85, the remaining transcripts were coded using the final category system (see Supporting Information 3). The text segments were paraphrased and translated into English. Finally, synthesised domains were included in Round 2's questionnaire or in the joint interpretation of both data sources (Figure 1, connecting and merging data). For Round 2's survey results, descriptive statistics were computed with statistical software R (version 4.3.2, RStudio Inc.). For selected items (Figure 1), consensus between respondents was determined. We defined consensus as 85% of participants with positive responses, that is, scale options 4 (‘tend to agree’) and 5 (‘totally agree’) [50]. We did not check for subgroup differences (e.g., between roles), as the exploratory nature of the study focused on identifying general patterns and themes across all stakeholders. For the mixed‐methods analysis, a sequential design was used to connect data during data collection [40]. A convergent design was employed to merge and compare selected results from both data sources via side‐by‐side displays [51, 52], helping us to validate findings or uncover inconsistencies [40, 47, 52].

## 3 Results

### 3.1 Participant's and PFACs' Characteristics

Fifteen PFACs were invited, with six agreeing to participate eventually. A total of 19 participants from the six PFACs across Germany (all University Hospital Settings; East: 3; West: 1; North: 1; South: 1) took part in the data collection. All 19 participants completed both Delphi rounds, resulting in a 100% response rate for each round and no participant dropouts at any stage of the process. Characteristics of participants and respective PFACs are depicted in Table 1. Sixteen respondents were patient/family stakeholders, and three came from a healthcare organisation/hospital. All PFACs had an oncological focus and were affiliated with a university hospital, respectively, often as part of a local or regional Comprehensive Cancer Centre. In approximately half (53%) of the cases, the PFAC consisted of 5–10 stakeholders and met quarterly.

### 3.2 Engagement and Functions of PFACs (T2—Mixed‐Methods Results)

Table 2 presents detailed mixed‐methods findings and contradictions regarding PFAC engagement, PS focus and PFAC roles. Additionally, illustrative quotes are provided in Supporting Information 4 (Table A). Levels of PFACS engagement and participation rights varied considerably across institutions and topics. While engagement in research activities (e.g., co‐developing study materials) was often perceived as active and structured, engagement in healthcare‐related topics was perceived as less consistent and limited in scope. This discrepancy was attributed to varying perceptions of actual degrees of participation, ranging from the level of ‘information’ to ‘shared leadership’ and from ‘very good’ to ‘insufficient’. Although respectful interaction was reported repeatedly, the impact of PFACs' input in decisions often remained uncertain. Participants expressed a desire for more direct and transparent involvement, alongside some concerns about unclear expectations or overburdening. So far, PS has not been an issue for PFACs and was unfamiliar to many. Nevertheless, participants emphasised a stronger PS focus and highlighted the need for access to clinic‐specific information to enable meaningful contributions. Interviewees outlined a broad range of potential roles and functions, from supporting individual patients to participating in strategic planning or quality processes. Across all areas, participants emphasised the importance of clearly defined roles, responsibilities and communication pathways to enable effective engagement.

**Table 2:** Side‐by‐side display with mixed‐methods results regarding PFACs' engagement, their focus on PS and roles and functions of PFACs.

Theme	Qualitative results (paraphrased)#	Quantitative results	Count, *n* (%)	Merged results
**Level of engagement and right of participation of PFACs**	Cooperation between PFACs and clinics was perceived as respectful and appreciative. The levels were at least not explicitly distinguished. Some felt that the voice of the PFAC was actively listened to, but it was mostly unclear to what extent it influenced the decision‐making process. Experiences of shared decision‐making, or where the practical influence of the PFAC was seen, tended to relate to research projects (e.g., formulating lay language for participant information). It was also felt that PFACs often lack the information and insight (e.g., PS data and internal clinical processes) to be able to participate actively. Patient representatives would like to have a more intensive exchange here. They also wanted to be involved directly, without having to go through several people/institutions. In the future, PFACs could also be more engaged at the structural level of care, with an active voice or vote, to represent the patient perspective there as well as in research. In addition, PFACs would like more feedback on the extent to which their voice has been considered in the decision. At the same time, however, some participants said that patient representatives should be protected from taking on too much responsibility or that giving them equal rights to participation would turn them into functionaries and lead to chaos in the clinic.	**Current level of PFAC engagement**		PFAC engagement varies widely by topic and location. PFACs are consulted, involved and actively listened to, but the ultimate impact of their opinions is often unclear. The level of engagement in research appears to be higher than in healthcare services. Collaboration with healthcare institutions is valued more in terms of respect and appreciation than in terms of actual influence and engagement. In the future, PFACs would like to have a greater right of participation in the provision of care and better and deeper access to information, figures and processes to be able to participate actively.
		The PFAC is informed about aspects of care, organisation, policy and so forth (‘informed’).	4 (21.1)	
		The PFAC is consulted on aspects of care, organisation, policy and so forth (‘consulted’).	4 (21.1)	
		The PFAC is involved in the development of care, organisation, policy and so forth (‘actively involved’).	6 (31.6)	
		The PFAC can co‐determine levels of care, organisation, policy and so forth on an equal basis (‘partnership and shared leadership’).	3 (15.8)	
		None of the answer options apply.	2 (10.5)	
		**Right of participation of the PFAC**		
		*How would you rate the current right of participation of the PFAC?*		
		1—very good	2 (10.5)	
		2—good	3 (15.8)	
		3—satisfactory	7 (36.8)	
		4—adequate	3 (15.8)	
		5—poor	1 (5.3)	
		6—insufficient	1 (5.3)	
		No opinion/unsure	2 (10.5)	
		*I think that the PFAC should have more rights of participation in the future.* *		
		Totally agree	5 (26.3)	
		Tend to agree	9 (47.4)	
		No opinion/unsure	4 (21.1)	
		Tend to disagree	1 (5.3)	
**Patient safety as a focus of PFACs**	PS has not been an explicit topic for PFACs so far. For many participants, PS was previously an unfamiliar concept, but PS was certainly addressed subliminally in some topics (e.g., the establishment of patient guides). PS was considered something that PFACs can and do want to have a right of participation. In the future, it was stated that PFACs could take on a kind of ‘watchdog role’ by sharing their PS experiences with themselves or other patients that the clinic is not aware of. Additionally, participants thought that PFACs need information on safety‐related data and cases from the clinic (e.g., on CIRS data) to focus on PS and to actively participate.	**PFAC's focus on PS**		PS has rarely been an issue and an unfamiliar concept for PFACs. PFACs see PS as an area they would like to focus more on in the future and see themselves as well‐suited to do so. A prerequisite for active engagement in PS is that PFACs have access to general and clinic‐specific safety‐related data and concepts.
		*I think that topics regarding PS have been sufficiently discussed so far.* *		
		Tend to agree	2 (10.5)	
		No opinion/unsure	4 (21.1)	
		Tend to disagree	11 (57.9)	
		Totally disagree	2 (10.5)	
		*I think that the PFAC should focus more on PS topics in the future.* *		
		Totally agree	5 (26.3)	
		Tend to agree	14 (73.7)	
**Roles and functions that PFACs could take on or assume**	The definition of the tasks and role of a PFAC and their individual stakeholders, as well as clear agreements on responsibilities and cooperation with the clinic, were expressed to be essential prerequisites for any work of PFACs. In addition to a ‘watchdog role’ for PS, PFACs could be involved in quality management processes and management committees (e.g., insight and evaluation of praise and complaint management data). Additionally, a PFAC may be involved in admissions or discharge management (e.g., by developing admissions guides for patients) or the creation of patient guides. It was also suggested that PFAC stakeholders should be present during individual consultations to bridge language barriers or knowledge gaps between the patient and the physician. PFACs are also seen as suitable for participating in the development of questionnaires, for example, as part of psycho‐oncological screening, or for developing profiles for research projects. Community outreach was another area in which PFACs are involved.	**Tasks that PFACs could assume** +		Before PFACs can be actively engaged, internal and external understanding of the roles of the entire PFAC and individual PFAC stakeholders must be explicitly defined. This includes defining the responsibilities and specific tasks of the PFAC and its stakeholders (not a merged result, only qualitatively). PFACs can and want to be engaged in many direct and overarching healthcare and PS issues. Currently, PFACs are mainly involved in research, but they can imagine engaging in healthcare in many areas.
		Representation of the patient perspective	19 (100)	
		Collaboration in research projects	19 (100)	
		Project work for patient care	18 (94.7)	
		Work in aftercare and discharge management	18 (94.7)	
		Engagement in complaint management	18 (94.7)	
		Public relations	17 (89.5)	
		Engagement in QRM	16 (84.2)	
		Engagement in management committees	15 (78.9)	
		14 (73.7)		

*Note:* N = 19.

* Likert scale 1–5 ‘totally disagree’ to ‘totally agree’, only response options that received at least one vote are shown.

^+^ Summary of how many participants chose 4 (‘tend to agree’) or 5 (‘totally agree’) on a 5‐point Likert scale.

^#^ Illustrative quotes of the qualitative results can be found in Supporting Information 4 (Table A).

### 3.3 Needs of PFAC Stakeholders for Future Interventions (T2—Delphi Results)

Based on Round 1's interview data, a total of 17 competency domains (9 related to healthcare safety and quality and 8 to communication) to be addressed in future educational interventions were extracted. In Round 2, we observed high agreement for 10 domains (with 5 for each competency type). All domains with corresponding percentages of agreement are presented in Table 3.

**Table 3:** Delphi results on PFAC needs and competencies for future interventions in PS.

Needs and competencies	Agreement
	*N* = 19, *n* (%)
*Types of competencies in healthcare safety and quality*	
Fundamentals of PS, error occurrence and error prevention	**19 (100)**
Knowledge of working in other (patient) organisations (e.g., self‐help groups)	**19 (100)**
Knowledge of hygiene measures and hygiene management (e.g., personal hand hygiene before patient contact and internal hygiene concepts)	**18 (94.7)**
Legal basis and requirements for PE and PFACs	**18 (94.7)**
Knowledge of quality and risk management	**17 (89.5)**
Knowledge of internal clinical procedures and processes (e.g., documentation of patient data and communication between different departments)	16 (84.2)
Knowledge and analysis of data relevant to PS (e.g. from error reporting systems)	16 (84.2)
Knowledge of the development and implementation of medical studies (e.g., methodological approach and interpretation of research results)	16 (84.2)
Knowledge of data protection and data security (in care and research)	16 (84.2)
*Type of competencies in communication*	
Clear and respectful manners (e.g., active listening, speaking out and actively asking questions)	**19 (100)**
Plain and precise communication of information	**18 (94.7)**
Objectivity and neutrality in the communication of complaints and problems	**17 (89.5)**
Proactive communication of constructive feedback	**17 (89.4)**
Acting and communicating in ways that serve the greater good	**17 (89.4)**
Communicating with regard to context and role (e.g., differences between internal and external exchange)	16 (84.2)
Diplomatic competencies and constructive discussion competencies	16 (84.2)
Linguistic fluency	15 (78.9)
*Level of competencies in healthcare quality and safety*	
Knowing and understanding PS concepts	11 (57.9)
Using PS concepts	5 (26.3)
Developing and evaluating PS concepts	2 (10.5)
*Level of competencies in communication*	
Knowing and understanding communication models	16 (84.2)
Using communication models	12 (63.2)
Developing and evaluating communication models	6 (31.6)
*Approx. duration for a theoretical part (e.g., e‐learning)*	
< 60 min	8 (42.1)
60–120 min	7 (36.8)
120 min up to half a day	3 (15.8)
Half a day to a whole day	1 (5.3)
*Approx. duration for a practical part (e.g., workshop)*	
120 min up to half a day	7 (36.8)
60–120 min	6 (31.6)
Half a day to a whole day	4 (21.1)
Not sure	2 (10.5)

*Notes:* Items had to be rated on a 5‐point Likert scale. Here is a summary of how many participants chose 4 (‘tend to agree’) or 5 (‘totally agree’). Consented items (≥ 85% on Likert scale items 4 or 5) are shown in bold.

Abbreviation: PFACs, patient and family advisory councils.

#### 3.3.1 Competencies in Healthcare Safety and Quality

Participants deemed fundamentals of PS, error occurrence and prevention (100% agreement) as highly relevant topics for future interventions (e.g., interviewee INT#4: ‘*What […] does patient safety mean? So that we really start by communicating the basics again*.’; INT#1: ‘*So not this ‘[…] I have a strange feeling about it’, […] but that you can really explicitly name the (risk) […] that goes with it*.’). Furthermore, knowledge about working with other (patient) organisations and networks (e.g., self‐help groups or other PFACs) was unanimously mentioned (e.g., INT#8 ‘*But it's good to meet […] with other PFACs […] and say, ‘What do you actually do […], how are you equipped, what options do you have? Yeah, what kind of problems do you have?' That would be a networking meeting*.’). In a similar statement, people who are also ‘*organized in self‐help [groups] have better prerequisites and more expertise in the subject than someone who is coping with his/her illness alone’* (INT#2). High agreement was also obtained on knowledge of hygiene measures and management (e.g., internal hygiene concepts) (94.7%), as PFACs could actively contribute to development and implementation of such approaches or the analysis of hygiene‐related reported incidents (e.g., INT#13: ‘*Then we would just need comprehensive information on hygiene measures, e.g., who is the representative? And to be informed about it and also to monitor these hygiene measures*.’). Surveyed PFAC stakeholders felt unaware of the legal basis and requirements for PE (94.7%). Potential consequences included advocating less for themselves and their rights or being less able to justify their engagement within clinics (e.g., INT#6: *‘I won't get any more research funding if I don't involve patients. […] But what about, for example, […] care processes? Is there one? Does the PFACs have to be involved?*’). Similar to above‐mentioned concepts in hygiene, patient representatives stated that increased knowledge about both general and subject‐specific concepts and processes of quality and risk management (89.4%) would be helpful (e.g., INT#9: *‘Sometimes I think I don't have enough insight at the macro level, […] at the organizational level of the clinic. I think I sometimes lack the insight to be able to participate and contribute well in audits and these quality management processes, for example.’*). Concerning subjectively appraised PS competency levels, 42.1% of participants indicated that they do not know or understand PS concepts, 73.7% stated that they cannot use safety concepts and 89.5% stated that they cannot develop or evaluate safety concepts (Table 3).

#### 3.3.2 Competencies in Communication

Regarding communication competencies, all participants emphasised social etiquettes in terms of clear and respectful manners, that is, active listening, speaking out and asking questions (e.g., INT#8: ‘*This is a basic requirement for communication, that you treat each other kindly, respectfully, that you listen, that you let others finish*’). Plain communication (e.g., use of patient‐oriented language) was also deemed a highly relevant topic (94.7%) (e.g., INT#3: ‘*[…] A language that lay people can understand. […] That's a basic requirement if the other person is to feel included […] and not get bored or decide “I don't understand this anyway, I'm just going to stop trying.” So you have to accommodate the PFAC stakeholder, the lay person, and you also have to have enough time and give them the confidence to ask questions*’). Further raised were communication about complaints and problems and proactive communication of constructive feedback (both with 89.5%) (e.g., INT#14: ‘*That you have to be careful with them and […] still say what you want […] and how you then manage to find a level of communication that is not hurtful’; INT#10: ‘Training how to deal differently with the clinic director, how to react to a rejection’)*. Participants also rated high relevance for learning to communicate in ways that serve the greater good (89.4%) (e.g., INT#11 ‘*[…] What can I do to make it better for the next patients? […] That has a bit to do with empathy, with the common good, I think. That's where I see the main purpose of the PFAC*’*; INT#7 ‘But I also think that over time they […] realize that it is no longer appropriate to tell so much about their own history, but that they simply represent a community’)*. Concerning individual appraisal of competency levels, 15.8% of participants indicated that they do not know or understand communication models, 36.8% stated that they cannot use communication models and 68.4% indicated that they cannot evaluate communication models (Table 3).

#### 3.3.3 Training Formats and Methods

Table 3 presents results on participants' responses who were requested to rate favourable durations for intervention modules. They indicated that a theoretical part of an intervention (e.g., through e‐learning) should not exceed 120 min (78.9% voted for ‘less than 60’ or ‘60 to 120 minutes’), whereas a practical part of an intervention (e.g., as workshop) could range from 60 min to a whole day.

The qualitative data showed that the desired formats and methods for future interventions vary and depend on many factors. Participants suggested modularising possible interventions (e.g., INT#19: *‘[…] When you look at how many subtopics there are […]? Does it make sense to modularize this, or can you do a multi‐day workshop or whatever?*’). Concerning mode of intervention, statements differed depending on the topics covered, number of participants, their individual capacities and time of the week (e.g., INT#19: *‘A very important point is that most of the patient representatives are patients themselves, with limitations, fatigue syndrome or other effects of the disease, which also means that they are no longer fully functional’;* INT#1: *‘So I'm struggling between online and in‐person. […] Practically it is […] better online. I think it's more effective and especially with the networking aspect, it's in‐person, yes’)*. Additionally, the specific engagement of PFACs in PS issues was requested, for example, through dissemination of best practice examples (e.g., INT#13: ‘*What […] best practice projects can we learn from? […] What do hospitals do? What are the critical issues for patients? We can also contribute to that. […] And when it comes to dialogue and cooperation, I would like to see a workshop with patient representatives and hospital management on expectations, obstacles [and] potential for improvement*’).

### 3.4 Factors Influencing PFACs' Work (T1—Qualitative Results)

Round 1's interviews revealed several key factors that influence the work of PFACs, which are outlined below. Illustrative quotes for each theme are provided in the Supporting Information 4 (Table B).

#### 3.4.1 Recruitment and Personal Requirements of PFAC Stakeholders

Recruiting new members for PFACs is challenging, as many patients face personal or health‐related constraints that may affect their ability to participate regularly as well as the expected continuity of their contributions. Further difficulties for recruitment were anticipated, yet stakeholders stated that closer collaboration with clinics could facilitate those efforts to involve patients or representatives.

#### 3.4.2 Resources

A recurring theme was the lack of resources, which is a significant barrier to effective PFAC work, particularly in terms of *financial support*. While hospitals were generally willing to implement recommendations from PFACs, there was reluctance to allocate substantial funds. The voluntary nature of PFAC work, often carried out by those with limited income (i.e., retirees and part‐time workers), added complexity. There was a stark contrast between the expectation of adequate compensation and the actual situation of PFAC members. In terms of *human resources*, participants highlighted the need for a designated contact person within the hospital to improve communication, clarify responsibilities and ensure better integration of PFACs. *Training opportunities* offered by broader networks (e.g., from the National Center for Tumor Diseases) were appreciated, but limited resources often hindered additional training. Strengthening existing *networks* was proposed as a means to better advertise existing programmes and participation opportunities and promote exchange between groups. Interviewees also suggested that individual *technical equipment* for home use could facilitate online participation in meetings and training. Basic on‐site equipment, such as projectors and microphones, was generally available. Lastly, *physical space* was also highlighted as a prevailing challenge. While meeting rooms were usually available, needs for a permanent on‐site presence were emphasised, such as designated rooms for regular office hours where patients can seek support.

#### 3.4.3 System‐Related Factors

Participants emphasised structural and organisational factors affecting PFAC's work. A key issue was the perceived *imbalance between ‘research PFACs’ and ‘routine healthcare PFACs’*. A key finding was the perceived disparity in available resources: while research PFACs often benefit from better financial compensation and training opportunities due to the prerequisite of PE for funding, this support is typically absent in care‐oriented PFACs. This imbalance raised questions about the fairness of resource allocation and led to calls for more equal conditions. Participants also expressed a desire for greater *insight into care and management processes*, such as access to inpatient rooms and complaint management, to better understand care standards and identify PS issues. Additionally, the *organizational culture regarding PE* and recognition of the PFACs among staff and patients remained limited, and increasing recognition within the organisation was deemed essential.

## 4 Discussion

Effectively engaging patients in the promotion of PS across all levels of healthcare is a major challenge. This study explored the needs of PFAC stakeholders regarding PS and communication competencies (primary objective), their engagement and roles and factors influencing PFACs' work (secondary objective). We used a Delphi‐consensus approach, combined with an exploratory, sequential and convergent mixed‐methods design, enabling a multifaceted investigation. Our results revealed that PFAC stakeholders expressed needs for nine competencies in healthcare safety and quality and eight competencies in communication, with consensus on five key domains in each category. In healthcare safety and quality, participants highlighted knowledge in PS fundamentals, collaboration with patient organisations and networks, hygiene measures, legal requirements for PE and quality management processes. Regarding communication competencies, participants emphasised the need for respectful communication, plain and patient‐oriented language, handling complaints, providing feedback and communicating the greater good. Our findings further contribute important recommendations on future educational and training interventions.

With regard to our primary objective—identification of needs for competency improvement—our study provides specific contributions to the yet limited body of research on PE in safety issues. Previous studies have acknowledged PS as a general educational need [3, 13, 34], but they largely overlooked the specification of competencies or failed to consider PFAC stakeholders as a potential key group with particular characteristics and educational needs. Yet, this is highly relevant given their heterogeneous backgrounds and structural diversity [22, 31, 35]. Our study proposes specific competencies that PFACs may apply in healthcare safety and quality improvements, such as PS basics, hygiene and quality management [26, 53]. The identified needs may also reflect an underlying desire to move beyond mere formal engagement towards actual, well‐informed participation within a partnership, including broader insights into clinical data, workflows and organisational processes. Limited awareness of legislative frameworks governing PE points to a critical gap between formal mandates and practical implications. Although there are common frameworks, such as the patient (safety) rights charter [54, 55], these were not well known among surveyed stakeholders. Enhancing the visibility of such frameworks and integrating them into ongoing interventions might be an effective strategy [22, 56]. Our study systematically identified communication‐specific competency improvement needs of PFAC stakeholders, such as respectful communication, patient‐friendly language and communication for a common good. Consistent with previous research, our findings highlight that patients often strongly associate PS with communication experiences with care providers, particularly respectful, comprehensible and partnership‐based interaction [37, 57, 58]. PFAC stakeholders also wished to strengthen their own communication skills, likely reflecting the challenges of navigating hierarchies and asserting their role as equal partners in dialogue with hospital professionals, management and leaders [59]. Interestingly, participants rated their communication skills as relatively high, presumably because PFACs may attract individuals with strong verbal skills. PFACs expect effective communication in their collaboration with healthcare organisations and seek to strengthen their own skills, highlighting the role of personal skills in PFAC functioning [15, 60]. Preferences for training formats highlight the value of modular, flexible approaches and relatable best‐practice examples, supporting previous calls for participatory, tailored programmes [12, 29, 61].

Regarding the secondary objective, our study revealed a significant role ambiguity within PFACs, with unclear mandates and blurred responsibilities. This lack of clarity hinders PFACs from fulfilling their purpose and achieving meaningful engagement in PS and limits their potential for structural integration into healthcare organisations. To address this, clear role definitions and well‐established governance structures are essential to ensure that PFACs transition from symbolic participation to active, structural partners in PS initiatives. Moreover, our findings showed significant variation in PFAC engagement across issues and settings [5, 21], yet most participants expressed a strong desire for more substantial involvement, which is consistent with previous findings [62, 63]. Also in line with other research, engagement was often described in terms of participation, respect and appreciation rather than actual influence [22, 35]. This reinforces concerns that PFACs, especially in hierarchical clinical contexts, are often just formally (i.e., symbolically) involved rather than as active partners, structurally embedded in strategic decision‐making [59]. To date, PS has a minor role in PFAC work, with many participants unfamiliar with the concept, highlighting its insufficient integration into PE contexts [13, 14, 34]. Nevertheless, participants expressed a strong interest in contributing to PS efforts, and therein several potential PFAC roles were suggested. A shared understanding of roles is crucial for effective PE, yet unclear structures often impede meaningful involvement [7, 13, 16]. Limited access to institutional data and opaque governance structures were repeatedly identified as barriers to meaningful PFAC engagement [7, 26], emphasising the need for transparency and operational access to enable substantive contributions to safety work. Our results also shed light on contextual factors influencing PFAC engagement, such as impeding barriers, including limited resources, institutional constraints and recruitment challenges [13, 16, 64, 65]. In response to these challenges, our study provides practical recommendations, such as coordinating training opportunities through overarching PFAC networks across healthcare institutions. This could compensate local shortages of educational resources and allow for broader, equitable access to training opportunities. Finally, there was a strong call for greater equity between research‐oriented and care‐oriented PFACs, highlighting underlying systemic inequalities: research‐based PFACs benefit from project funding and academic infrastructure, while care‐oriented PFACs often rely on institutional goodwill. These imbalances result in lower visibility and influence for care‐oriented PFACs, limiting the transferability of successful engagement models and reinforcing systemic inequalities. Structural reforms are needed to create fair conditions for all PFACs.

### 4.1 Strengths and Limitations

This study has strengths and limitations that should be considered when interpreting the findings. A key strength is the combination of a Delphi design with two rounds and a complementary mixed‐methods approach, allowing for a multidimensional assessment of PFAC stakeholders' needs. The Delphi process facilitated the refinement of expert input [39, 50], while combining qualitative and quantitative data enhanced the depth and validity of the findings, leading to a more comprehensive understanding [40, 47, 52]. Moreover, the study was conducted in a participatory manner and with close alignment to the lived realities of the target group, thereby ensuring relevance and methodological sensitivity. However, the mere limitation to two Delphi rounds may have prevented a natural stopping point for new information, potentially limiting the emergence of further insights and nuanced topic distinctions [39, 43]. The preselection of topics based on prior qualitative assessments may have reinforced their perceived relevance, contributing to uniformly high importance ratings and limited differentiation. While this could suggest a consensus, it does not necessarily reflect a clear prioritisation of topics.

Standardised survey instruments could have improved comparability but were constrained by practicability and tool availability [66]. While participants interpreted the term PS in interviews differently and attributed high relevance to basic PS knowledge in the survey, 60% reported familiarity with PS and communication. This suggests varying interpretations of what PS competency entails and may also point towards potential misunderstandings of PS, conceptual ambiguities or our assessment approach. Furthermore, social desirability bias may have influenced the survey responses, leading to participants overstating their familiarity with or competence in PS‐related domains. In addition, the interviews were conducted before the surveys, which may have given participants time interim to further reflect on the topic, potentially further stimulating their familiarity with PS. Using both methods likely mitigated measurement bias, but observed discrepancies highlight the need for more standardised, clearly defined instruments—at least for the target population—to improve accuracy and minimise conceptual ambiguity. However, participants' unfamiliarity with PS suggests deeper needs for targeted educational interventions and increased PS awareness, emphasising the potential role of PFACs in preventing avoidable safety incidents [3, 6, 9].

The composition of the sample also poses potential limitations. Our oncology‐focused, German sample may limit external validity. Yet, several needs pertaining outside of oncology emerged, and findings align well with studies from non‐oncological care settings. The specific cultural and institutional contexts in which our study was conducted should also be considered when applying or transferring our findings to other settings. Additionally, many participants were active in support groups, reflecting the diverse backgrounds that PFACs intentionally bring together [19, 67]. Rather than representing a single perspective, this diversity shapes expectations for future interventions and can lead to different priorities and time conflicts. Moreover, low hospital representative participation likely reflects structural barriers, such as time constraints, staffing shortages and differing institutional PE priorities [13, 68]. Recruitment efforts included contacting PFAC coordinators to forward the study invitation, which may have been an additional limitation. In line with this, unclear roles occurred during the recruitment and survey, such as who coordinates PFAC engagement. Targeted measures, such as a designated liaison role for PFACs with institutional support, could facilitate engagement [69]. As stated in the ‘Methods’, exploration of subgroup differences (e.g., by role) was not an objective of this study. Additionally, the small sample size, particularly among hospital professionals, did not allow for meaningful comparisons of the perceived importance of competencies between patient/family stakeholders and hospital staff. Therefore, future studies should strive for larger and more balanced samples that allow for reliable subgroup analyses to determine differences between stakeholder perceptions of PFAC work and to explore potential differences in competency priorities between these stakeholder groups. Future research should also consider PFAC structures and dynamics to identify context‐specific support needs.

### 4.2 Implications for Healthcare Practice and Research

Concerning practical implications, first, roles, functions and participation rights of PFACs should be clearly defined and communicated to align expectations and support effective collaboration between PFAC stakeholders and healthcare institutions [7, 13, 22, 59]. However, our findings suggest that such clarity is often lacking in practice, in part due to the informal development of PFACs and the absence of well‐described institutional mandates. Role ambiguity not only hinders effectiveness but can also lead to tokenistic engagement. Therefore, efforts to define roles shall be well aligned with solid embedment in the hospital organisation and institutional accountability. Second, healthcare organisations should address the specific needs of PFACs to enhance their contributions, sustain engagement and support long‐term motivation and retention. When contributions are recognised and result in actual change, engagement is more likely to be sustained [9, 61, 63], while epistemic injustice, by dismissing patients' voice, can hinder their meaningful engagement in PS efforts [59]. Third, organisations should facilitate PFACs' access to quality management structures and institutional data to enhance transparency and enable meaningful participation in PS efforts, including structured onboarding of new stakeholders [22, 70]. However, integrating PFACs into care processes remains challenging due to limited resources, unclear governance and hierarchical structures that marginalise their role. Without leadership commitment and structural reform, PFACs risk tokenism and lack influence on PS. True and feasible integration requires operational access, clear governance, sustained funding and adjustments to workflows. Fourth, targeted educational interventions are necessary [29, 60]. Our findings provide a basis for designing such interventions, which should address the proposed content areas and structural barriers. These interventions should be modular and tailored to varying levels of prior knowledge, availability and accessibility. Training should also include best practice examples, support for PE and enable cross‐institutional learning through networking opportunities [22, 71]. Based on the identified needs and existing literature, we have developed a corresponding intervention as part of our PEPS‐3 project. Specifically, the findings of this study were used as part of a participatory approach at the level of active involvement to inform the development of a two‐module intervention (1. e‐learning and 2. workshop). Additional inputs, including insights from a rapid scoping review and stakeholder feedback, were also considered in the design. While the detailed development process of this intervention goes beyond the scope of this article, it aims to address the structural barriers and content gaps highlighted in the findings and will be reported and evaluated separately [72]. Fifth, to ensure equitable integration, the same favourable conditions should be established for research‐ and care‐oriented PFACs. Without long‐term resource allocation and commitment, such as funding for travel, meeting space, digital participation and reimbursement, contributions of care‐oriented PFACs will remain marginal, distorting the representation of patient needs [13, 64].

Concerning implications for research, our findings contribute to the scientific discourse on PFAC engagement in PS by particularly addressing both structural and methodological gaps. While PE is well established in research contexts, its role in routine care remains less defined. Oncology serves as a leading example, as PE is often part of certification requirements. However, integration in other medical specialties remains inconsistent. Future research should examine how institutional frameworks shape PFAC engagement in PS across specialties. It would also be interesting to conduct specific studies on how PFACs can support organisational safety initiatives such as incident reporting [10]. The under‐representation of hospital professionals in our study also highlights the need to further explore clinicians' availabilities and perspectives on the role of PFACs in PS, that is, understanding how care providers align daily workload with involvement in PFACs. Future research should aim to include a more diverse sample, particularly clinicians, to explore how PFACs can be integrated into diverse institutional contexts. Methodologically, the lack of standardised instruments to assess stakeholders' competencies and engagement limits comparability [66]. Finally, the study highlights the importance of a thorough evaluation of training and networking interventions. The needs identified provide a valuable basis for developing and testing capacity‐building interventions. Further research should also explore how different forms of patient representation, such as PFACs or self‐help groups, can be integrated into coherent PE strategies, leveraging their role in nominating representatives and fostering regional/national networking, to promote PS in clinical care.

## 5 Conclusion

This study provides novel insights into competency improvement needs of PFAC stakeholders in PS, including PS fundamentals, legal basis for PE and respectful communication. By identifying the specific competency areas alongside key implementation factors, our findings can inform the design of targeted, participatory training formats and respective organisational support strategies. To strengthen PFAC engagement, healthcare organisations should clarify roles, ensure structured integration into decision‐making and provide adequate resources. Greater alignment between research‐ and care‐based PFACs, as well as embedding PFACs into routine safety and quality structures, will be essential to improve feasibility and long‐term impact.

## Author Contributions

**Larissa Brust:** conceptualisation, data curation, formal analysis, investigation, methodology, project administration, writing – original draft, validation, visualisation. **Yannick Blum:** conceptualisation, data curation, formal analysis, investigation, methodology, validation, writing – review and editing. **Matthias Weigl:** conceptualisation, funding acquisition, methodology, project administration, supervision, writing – review and editing.

## Disclosure

During the preparation of this study, the authors used ChatGPT 4 to improve readability and language. After using this tool/service, the authors reviewed and edited the content as needed and take full responsibility for the content of the publication.

## Ethics Statement

The study was approved by the Ethics Committee of the University of Bonn (Ref. nr: 2024‐236‐BO). Data collection and processing was carried out in accordance with the Declaration of Helsinki, the European Union's General Data Protection Regulation (GDPR) and the German Federal Data Protection Act (BDSG).

## Consent

All participants of the study signed an informed consent.

## Conflicts of Interest

The authors declare no conflicts of interest.

## Supporting information

Supp 1 PreSurvey QualitativeInterview Guide T1.

Supp 2 Quantitative Survey T2.

Supp 3 Category system for qualitative content analysis.

Supp 4 Qualitative quotes for illustrative purposes.

## Data Availability

The data that support the findings of this study are available at the request of the corresponding author and are subject to ethics committee approval and relevant legal requirements. The data are not publicly available due to privacy or ethical restrictions.
