# Supplementary material for: Promoting Patient Safety Through Patient Engagement at the Organisational Level: A Delphi‐Based Needs Assessment Among Patient and Family Advisory Councils
Source: Health Expect. 2025 Jun 10;28(3):e70319. doi: 10.1111/hex.70319 (PMC12149985; doi:10.1111/hex.70319)
Supplement: Supplementary file 1 — Supp 1 PreSurvey QualitativeInterview Guide T1. [file HEX-28-e70319-s002.docx]

# **Supporting Information 1. Pre-Survey and Qualitative Interview Guide (T2)**

## Pre-Survey

**1. Pseudonym**

Please enter your study code that you have been assigned by us here:

**2. Personal data**

Please enter your age:

Years

For how long have you been a member of the patient and family advisory council (PFAC)?

Months

Which groups of people on the PFAC do you belong to?

Please choose the corresponding category (multiple options possible).

|  |  | Affected persons/patients |
| --- | --- | --- |
|  |  | Patient representatives or members of patient organizations (please specify): |
|  |  | Family members |
|  |  | Doctors |
|  |  | Nurses |
|  |  | Quality and risk management office representatives |
|  |  | Other caregivers |
|  |  | Moderators |
|  |  | Other, specifically: |

How many hours per month do you dedicate to the PFAC?

Please choose the corresponding category

|  |  | Less than 5 hours per month |
| --- | --- | --- |
|  |  | 5 to 10 hours per month |
|  |  | 11 to 20 hours per month |
|  |  | More than 20 hours per month |

What motivated you to become a member of the PFAC?

Please choose the corresponding category (multiple options possible).

|  |  | Personal experience as a patient |
| --- | --- | --- |
|  |  | Initiating improvements in the healthcare system |
|  |  | Active participation in healthcare decision-making |
|  |  | Networking |
|  |  | Personal growth |
|  |  | Experiences of relatives |
|  |  | Professional activity in the PFAC |
|  |  | Other, specifically: |

**3. Organization and structure of the PFAC**

What organization is the PFAC you belong to affiliated with?

Please choose the corresponding category (multiple options possible).

|  |  | University Hospital |
| --- | --- | --- |
|  |  | Hospital |
|  |  | University |
|  |  | Health centre / medical care centre |
|  |  | Other, specifically: |

Which groups of people compose the PFAC to which you belong?

Please choose the corresponding category (multiple options possible).

|  |  | Affected persons/patients |
| --- | --- | --- |
|  |  | Patient representatives or members of patient organizations (please specify if possible): |
|  |  | Family members |
|  |  | Doctors |
|  |  | Nurses |
|  |  | Quality and risk management office representatives |
|  |  | Other caregivers |
|  |  | Moderators |
|  |  | Other, specifically: |

How often do the meetings of the PFAC take place?

Please choose the corresponding category.

|  |  | Several times a month |
| --- | --- | --- |
|  |  | Monthly |
|  |  | Quarterly |
|  |  | Half-yearly |
|  |  | Yearly |
|  |  | Unregularly, specifically: |

How many stakeholders are there in the PFAC to which you belong?

Please choose the corresponding category.

|  |  | Less than 5 members |
| --- | --- | --- |
|  |  | 5 to 10 members |
|  |  | 11 to 15 members |
|  |  | 16 to 20 members |
|  |  | Over 20 members |

How does the cooperation between the PFAC, to which you belong, and the healthcare organizations work?

The degree of engagement usually differs depending on the situation. Please select the category that applies most frequently.

|  |  | The PFAC is informed about aspects of care, organization, policy, etc. (‘informed’). |
| --- | --- | --- |
|  |  | The PFAC is consulted on aspects of care, organization, policy, etc. (‘consulted’). |
|  |  | The PFAC is involved in the development of care, organization, policy, etc. (‘actively involved’). |
|  |  | The PFAC can co-determine levels of care, organization, policy, etc. on an equal basis (‘partnership and shared leadership’). |
|  |  | None of the answer options apply. |

Does the PFAC to which you belong receive resources / support from the cooperating organization? If yes, which ones?

Please choose the corresponding category (multiple options possible).

|  |  | There is no support from the cooperating organization |
| --- | --- | --- |
|  |  | Financial support |
|  |  | Further education/training |
|  |  | Infrastructure for online services being used (mobile phone, tablet, PC, projector, etc.) |
|  |  | Rooms and other facilities |
|  |  | Expertise/consulting |
|  |  | Support in the expansion of networks and co-operations |
|  |  | Support in public relations work |
|  |  | Other, specifically: |

**4. Final page**

Thank you for your engagement and for taking the time to complete our survey. For the following steps, we will contact you shortly.

Yours sincerely,

The PEPS 3.0 project team of the Institute for Patient Safety.

## Interview Guide for Round 1

| Introduction & Explanations  Questions?  Provide information about the recording of the video and obtain verbal consent + Start Recording  Clarify the role of the participant | | |
| --- | --- | --- |
| **TOPIC** | **MAIN QUESTIONS** | **ADDDITIONAL QUESTIONS/ EXAMPLES (optional)** |
| **Engagement, roles, and functions of PFACs** | How do you experience the communication between the stakeholders of the PFAC and the healthcare organization? | - For example: respect, being taken seriously, being listened to, the right topic at the right time, respecting speaking time, conversation rules, ... |
|  | How do you experience the degree of engagement and right of participation of the PFAC in terms of patient safety (PS) in the healthcare organization? | - “informed”, “consulted”, “actively involved”, “partnership and shared leadership” - What could be improved to promote the engagement of PFACs in the field of PS? |
|  | What roles and tasks could PFACs take on to promote PS? | - Can you give specific examples of how PFACs can act on PS issues?   - speaking up, providing the patient's perspective, importance of inner/subjective experience, transparency, etc. - Why don't you see PFAcs as being able to promote PS? (if expressed that it does not matter) |
| **Knowledge/ competencies** | How do you estimate your competences and skills and those of the other stakeholders in the PFAC with regard to PS? | - In your opinion, what skills are required to effectively participate in and influence PS issues as a member of the PFAC? - For example: Recognising safety-relevant events/topics, key terms and concepts of PS and fields of action, relevance of the topic of PS and its significance for the patient's care, ... |
|  | How would you estimate your communication skills and abilities and those of the other stakeholders on the PFAC? | - In your opinion, what skills are required to effectively participate in and influence PS issues as a member of the PFAC? - For example: respect, taking each other seriously, listening, the right topic at the right time, keeping to speaking time, changing perspectives, rules of conversation etc. |
| **Influencing factors/ conditions** | What structures, resources, processes, or requirements would you like to see for the PFAC in terms of PS, dialogue and cooperation between PFAC and hospital staff? | - What aspects could be improved to promote engagement and participation? - For example: premises, skills training, time, technology, money, networking, … |
| **Intervention formats and methods** | How could we improve your knowledge and skills and those of the other stakeholders on the PFAC in the areas of PS, communication and dialogue between the PFAC and the healthcare organization? | - What would you expect from an educational program?   - on PS in general?   - to promote knowledge and skills in communication and dialogue between the PFAC and the healthcare organization? - Which formats and methods should not be used under any circumstances? - Focus on PS; example: workshop, structured instructions, online material, learning status surveys, literature, … - How should online programmes be designed in this respect? |
|  | What else is important to you with regard to the content, formats and implementation of the training/workshop program? | - For example: place, time, weekdays/weekends, number of units, group work, individual work, etc. |
| **Conclusion** | We now come to the end. Do you have any points that we should have addressed but didn't?  Is there anything else you would like to say? | - If you had one wish, what would you like to see in terms of promoting the engagement of PFACs to promote PS? |
| **Farewell & further procedure** | Thank you very much for your time and contribution. We greatly appreciate your input.  Stop recording.  Explanation of further procedure. | |
