# Supplementary material for: Promoting Patient Safety Through Patient Engagement at the Organisational Level: A Delphi‐Based Needs Assessment Among Patient and Family Advisory Councils
Source: Health Expect. 2025 Jun 10;28(3):e70319. doi: 10.1111/hex.70319 (PMC12149985; doi:10.1111/hex.70319)
Supplement: Supplementary file 2 — Supp 2 Quantitative Survey T2. [file HEX-28-e70319-s004.docx]

# **Supporting Information 2. Quantitative Survey (T2)**

**1.** **Pseudonym**

Please enter your study code that you have been assigned by us here:

**2.** **Engagement of PFACs**

How would you rate the current right of participation of the PFAC?

| 6 –  Insufficient | 5 –  Poor | 4 –  Adequate | 3 – Satisfactory | 2 –  Good | 1 –  Very good | No opinion / Unsure |
| --- | --- | --- | --- | --- | --- | --- |
|  |  |  |  |  |  |  |

I think that the PFAC should have more right of participation in the future.

| Totally disagree | Tend to disagree | No opinion / Unsure | Tend to agree | Totally agree |
| --- | --- | --- | --- | --- |
|  |  |  |  |  |

**3. PFAC’s focus on patient safety**

I think that topics regarding patient safety have been sufficiently discussed so far.

| Totally disagree | Tend to disagree | No opinion / Unsure | Tend to agree | Totally agree |
| --- | --- | --- | --- | --- |
|  |  |  |  |  |

I think that the PFAC should focus more on patient safety topics in the future.

| Totally disagree | Tend to disagree | No opinion / Unsure | Tend to agree | Totally agree |
| --- | --- | --- | --- | --- |
|  |  |  |  |  |

**4. Info page**

On the following pages you are asked to evaluate the content and methods for an educational and workshop format and to rate them according to your personal preference. The content of the format will include patient safety topics as well as communication and dialogue facilitation to promote co-decision making on patient safety topics. Your answers and those of the other participants will form the basis for an educational and workshop format (e.g., regarding the focus and selection of topics, their preparation and the formats used).

The possible answers in the individual questions are based exclusively on the statements we collected from the previous interviews. The program is planned in two modules. Module 1 is intended to provide theoretical knowledge and basic principles in the form of a course. Module 2 is designed as a workshop to transfer the theoretical topics into the practical work of your PFACs and to invite a collective exchange of ideas. In the following questions, you are sometimes asked to distinguish between Module 1 (education) and Module 2 (workshop).

**5. Roles and responsibilities**

I think that PFACs could be actively involved in the following topics and tasks.

|  | Totally disagree | Tend to disagree | No opinion / Unsure | Tend to agree | Totally agree |
| --- | --- | --- | --- | --- | --- |
| Individualised support for patients in hospital (for example, engagement of relatives in the treatment process, contact persons, counselling services, support for patients in consultations with doctors, patient café). |  |  |  |  |  |
| Work in aftercare and discharge management (for example referral of patients to self-help groups, engagement in discharge management concepts). |  |  |  |  |  |
| Representing a diverse patient perspective to organizational staff. |  |  |  |  |  |
| Project work for patient care (for example, co-design and testing of informational material, internal clinic concepts and guidelines, acquisition of project funding). |  |  |  |  |  |
| Insight into and engagement in quality and risk management (for example, insight into error reporting systems) |  |  |  |  |  |
| Praise and complaint/conflict management (for example, mediation between hospital staff and patients, feedback on communication). |  |  |  |  |  |
| Collaboration in research projects (for example, co-development of the research question, advice on the methodological approach, analysis and evaluation of data, development of research profiles). |  |  |  |  |  |
| Public relations and representation (for example, healthcare organization representation at congresses, external communication, including project results). |  |  |  |  |  |
| Engagement in various management committees (for example, tumour boards, nursing service and executive committee meetings). |  |  |  |  |  |

**6. Competencies in Healthcare Safety and Quality**

I think that the following skills should be given a high priority in an educational program.

|  | Totally disagree | Tend to disagree | No opinion/Unsure | Tend to agree | Totally agree |
| --- | --- | --- | --- | --- | --- |
| Fundamentals of patient safety (PS), error occurrence and error prevention |  |  |  |  |  |
| Knowledge and analysis of data relevant to PS (e.g. from error reporting systems) |  |  |  |  |  |
| Knowledge of hygiene measures and hygiene management (e.g., personal hand hygiene before patient contact, internal hygiene concepts) |  |  |  |  |  |
| Knowledge of internal clinical procedures and processes (e.g., documentation of patient data, communication between different departments) |  |  |  |  |  |
| Knowledge of quality and risk management |  |  |  |  |  |
| Knowledge of the development and implementation of medical studies (e.g., methodological approach, interpretation of research results) |  |  |  |  |  |
| Knowledge of data protection and data security (in care and research) |  |  |  |  |  |
| Legal basis and requirements for PE and PFACs |  |  |  |  |  |
| Knowledge of working in other (patient) organizations (e.g., self-help groups) |  |  |  |  |  |

**7. Competencies in Communication**

I think that the following skills should be given a high priority in an educational program.

|  | Totally disagree | Tend to disagree | No opinion/Unsure | Tend to agree | Totally agree |
| --- | --- | --- | --- | --- | --- |
| Linguistic fluency |  |  |  |  |  |
| Plain and precise communication of information |  |  |  |  |  |
| Clear and respectful manners (e.g., active listening, speaking out, actively asking questions) |  |  |  |  |  |
| Objectivity and neutrality in the communication of complaints and problems |  |  |  |  |  |
| Diplomatic competencies and constructive discussion competencies |  |  |  |  |  |
| Proactive communication of constructive feedback |  |  |  |  |  |
| Acting and communicating in ways that serve the greater good |  |  |  |  |  |
| Communicating with regard to context and role (e.g., differences between internal and external exchange) |  |  |  |  |  |

**8. Level of training**

Please assess the following statements regarding your level of knowledge and competencies for your work on the PFAC.

Your assessment will help to determine the appropriate level of a program.

|  | Totally disagree | Tend to disagree | No opinion / Unsure | Tend to agree | Totally agree |
| --- | --- | --- | --- | --- | --- |
| I know and understand the basic concepts of patient safety. |  |  |  |  |  |
| I use patient safety concepts. |  |  |  |  |  |
| I develop and evaluate safety concepts. |  |  |  |  |  |
| I know and understand basic models and strategies of effective communication. |  |  |  |  |  |
| I use models and strategies of effective communication. |  |  |  |  |  |
| I develop and evaluate models and strategies of effective communication. |  |  |  |  |  |

**9. Time required for e-learning and workshop**

For the different modules I would like to have an approximate time effort of...

The times given refer to the pure working duration. Breaks can be taken flexibly and are not included in the amount of time listed here (for example, you can work for 30 minutes on four different days during the educational program).

|  | Less than 60 minutes | 60 to 120 minutes | 120 minutes to half a day (4 hours) | Half a day to a whole  day (4 to 8 hours) | No opinion /  Unsure |
| --- | --- | --- | --- | --- | --- |
| Theoretical e-learning module (online, individual completion). |  |  |  |  |  |
| Practical workshop in presence (with your PFAC members and healthcare representatives). |  |  |  |  |  |

If you have any other comments, suggestions or wishes for the educational and workshop format that have not yet been addressed in this questionnaire, you have the opportunity to leave a comment here.

For the educational and workshop format I also wish:

**10. Final page**

Thank you for your engagement and for taking the time to complete our survey. For the following steps, we will contact you shortly.

Yours sincerely,

The PEPS 3.0 project team of the Institute for Patient Safety.
