# Supplementary material for: Promoting Patient Safety Through Patient Engagement at the Organisational Level: A Delphi‐Based Needs Assessment Among Patient and Family Advisory Councils
Source: Health Expect. 2025 Jun 10;28(3):e70319. doi: 10.1111/hex.70319 (PMC12149985; doi:10.1111/hex.70319)
Supplement: Supplementary file 3 — Supp 3 Category system for qualitative content analysis. [file HEX-28-e70319-s003.docx]

# Supporting Information 3. Category System for Qualitative Content Analysis

| **Main category** | **Content of the main category** | **Subcategory** | **Content of the subcategory** |
| --- | --- | --- | --- |
| **Engagement, roles, and functions of PFACs** | - roles and functions that PFACs could take on or assume - current and desired PFAC’s engagement in care and patient safety issues - engagement in measures, processes, decisions, and networks to support shape research and care   e.g., co-design of treatment processes, development of patient information, evaluation of quality of care, collaboration in research projects, engagement in audits and congresses, contact persons for patients | **Type of engagement and collaboration** | - channels of communication and interaction - organization of meetings and interaction - types of engagement (i.e., information, involvement, partnership) - satisfaction with engagement - transparency of processes (access to information, feedback on enquiries and suggestions) |
|  |  | **Topics and areas of engagement** | - care processes, patient safety - structural and quality processes - research projects |
|  |  | **Other** | Content that belongs to the main category but does not fit into subcategories. |
| **Individual knowledge, competencies, and requirements** | - knowledge, skills and experience that enable comprehensive and effective engagement in patient safety and communication issues and dialogue between the PFAC and the healthcare organization   e.g., communicating clearly, changing perspectives, applying rules of dialogue, knowledge of safety measures, and error occurrence, health status, previous training, knowledge of system-related conditions | **Healthcare safety and quality-related** | - patient safety. care and quality management, hygiene measures - knowledge of system-related conditions or other organizations - legal basis and requirements for patient engagement (in patient safety) - medical research projects - data protection and security |
|  |  | **Communication-related** | - ability to work together, constructive communication and dialogue, application of communication techniques, plain communication - context and role/common good |
|  |  | **Person-related** | - ability to abstract problems - influencing symptoms - openness and commitment - background and previous experience |
|  |  | **Other** | Content that belongs to the main category but does not fit into subcategories. |
| **Influencing factors and conditions** | - external, organizational, legal, and cultural conditions that influence the work of PFACs   e.g., structure of the healthcare system, legal requirements for patient engagement, internal healthcare organization guidelines, communication culture, resources and further training measures | **Resources** | - financial support, human resources - physical space, technical equipment - access to training and further education |
|  |  | **System-related conditions** | - research- vs care-oriented PFACs, legal requirements - support from healthcare organization - clear structures and responsibilities for engagement - organizational culture of cooperation and patient engagement - insight into care and management processes |
|  |  | **Other** | Content that belongs to the main category but does not fit into subcategories. |
| **Intervention content, formats, and methods** | - education and training content, measures, and formats that participants explicitly desire to improve competences, skills, dialogue and engagement as part of an intervention   e.g., specific teaching methods, group work, educational games, time frames, structure | **Contents** | - patient safety; communication strategies and techniques - measures to promote the engagement of PFAC - best practice examples |
|  |  | **Formats and methods** | - online/in-person/hybrid, modularization - individual vs group work, number of participants - duration and frequency (i.e., continuous training, one-time event, time of day) |
|  |  | **Other** | Content that belongs to the main category but does not fit into subcategories. |
| **Other** | Content that does not fit into one of the main categories. | NA | NA |
