# Supplementary material for: Promoting Patient Safety Through Patient Engagement at the Organisational Level: A Delphi‐Based Needs Assessment Among Patient and Family Advisory Councils
Source: Health Expect. 2025 Jun 10;28(3):e70319. doi: 10.1111/hex.70319 (PMC12149985; doi:10.1111/hex.70319)
Supplement: Supplementary file 4 — Supp 4 Qualitative quotes for illustrative purposes. [file HEX-28-e70319-s001.docx]

# Supporting Information 4. Qualitative Quotes for Illustrative Purposes

## **Table A. Qualitative quotes for PFACs’ engagement, their focus on patient safety, and roles and functions of PFACs**

| **Theme** | **Illustrative Quote** |
| --- | --- |
| Level of engagement and right of participation of PFACs | INT#16: ‘*I would say it is still in its early stages. (...) So, in other words, (...) efforts are being made, but it has not really taken off that patient advisory councils are heavily involved. You can have a whole range of involvement with these patient advisory councils, from tokenism to really good collaboration. And we are still in the early stages of that.’* |
|  | INT#6: ‘*So I thought, one says, that's actually 100%. So I can say where I want something, where I have an idea. So it's not blocked, on the contrary, it's really welcome [...]. It was about revising research, profiles and so on, and it was immediately taken up and implemented. So I thought that was really remarkable. As far as the decision is concerned, I have to say that I can't judge that, because I still don't understand exactly what the decision-making structures are for what happens at the end of the day in the clinic in the daily routine of the clinic, and what the formal processes are, so to speak, where a clinic actually has to involve the patient advisory council in order to be able to make a decision at the end of the day? I'm a little bit overwhelmed.’* |
|  | INT#6: ‘*So you also need data to be able to influence things. Let's say we want to know if a molecular tumor board is well attended, if it's well received, how it's going. We need data for that. So, we are being treated well. But you can't just stay on the surface. You have to go deeper into an issue. And that hasn't happened yet.’* |
|  | INT#12: ‘*We participate in the council meetings [...]. But we always have a block in which we can discuss, in which we can present our issues, ask questions, bring in ideas. [...] And then we can only either complain or discuss, but not decide.’* |
|  | INT#7: ‘*But I have been here for many years now, I have ten years of experience, and an incredible amount has happened. In the beginning, there was actually a little bit of a feeling that, um, it might be a token thing. The patient advisory council might decorate the hospital quite well, there were posters and flyers and so on, but they weren't really active and you weren't necessarily involved. And I think that has changed a lot in the last few years. So that the patient's point of view is really in demand and the patient's voice is wanted and also that the patient is really invited to meetings and committees and also to the director's office during office hours. Or rather, that the patient now participates in the meetings and so, um, there has been a great development.’* |
|  | INT#4: ‘*The patient advisory council members are always very focused. They really dig into the issues. We send some things out in advance so that the council members have time to study the topics. And then we always get very valuable feedback and assessments from the council members. And we always try to integrate that, either into scientific projects or into structural aspects or public relations issues.’* |
|  | INT#3: ‘*I can say that I not only have a say in this area, but that I can also help formulate the decisions, that I can therefore always contribute our point of view, our point of view from the patient's perspective, and that my opinion is always heard.’* |
|  | INT#19: ‘*It is not yet so common or standard for a patient advisory council to be deeply involved in the decision-making process. Rather, it is often the case that I have also checked the box for the council to be more informed about what is going on in the clinic, so that we can participate in certain events and present ourselves and be there. But this real involvement, that we as patients can even come up with ideas on how we can improve something or change something. We are still far away from that.‘* |
|  | INT#12: ‘*So no decision, no say, although if issues arise in this field, we are certainly heard. But of course we don't really have a right to vote.’* |
|  | INT#6: ‘*And what I experienced was a great appreciation for me as a patient. When I actually got involved in the patient advisory council, I found the discussions to be absolutely equal. I also found that remarkable. I was also astonished to have the feeling, especially from the clinical side with scientific know-how, that I was taken seriously as a patient with my questions or concerns or as a representative of the patient advisory council. And what really impresses me to this day is the commitment to communication. When agreements are made, they are kept.‘* |
|  | INT#15: ‘*Unfortunately, [I often find that communication is] very slow. [...] So that it takes a very long time for someone to reply, or [...] that our presence is often not really desired.’* |
|  | INT#13: ‘*Communication with the clinic is excellent. [...] We take great care to ensure that we are involved.’* |
|  | INT#9: ‘*I experience it as totally warm and appreciative, really very open, that in theory you can contribute any concern or idea that you have as a member. So there is always room for that. Room is always consciously actively offered for that.’* |
|  | INT#2: ‘*So, requests to us are relatively rare, but of course we have a lot of wishes and also points of criticism where we would like to have better information and actually also a direct involvement not through two or three people.’* |
|  | INT#12: ‘*But first and foremost, we need to get feedback from the patients as well. What is the problem? We don't get that from the doctors as much. [...] We would like to have a little more insight into the finances. So far, nothing has come of that either. So we would like to see a more intensive and also a little more honest and open exchange.’* |
|  | INT#16: ‘*Then there's a tug-of-war among the patient representatives, then it's the same as with the other officials. What do you think would happen if I had a vote? There would be a lot of backstabbing. We have to be given certain rights, like knowing how to intervene in the process, the right to appeal, to object, or something like that. But anything else would make us functionaries, looking after our own interests. We can no longer do that. So I don't think much of it.’* |
|  | INT#7: ‘*No, but I always want to warn or protect patient representatives from taking on too much responsibility.’* |
| Patient safety as a focus of PFACs | INT#6: ‘*So I think that we, as patient representatives, could be something of a watchdog in that role. So that we look at everything that is discussed in the patient advisory council and see where it might have an impact on patient safety? (...) Then I think we could also be shapers and designers in the patient advisory council. In other words, we can be proactive in bringing in aspects from the patient's point of view that are related to patient safety and that may not even come to the fore from a clinical point of view.’* |
|  | INT#16: ‘*If we were familiar with such concepts, then we could of course use our experience to say, ‘Oh, we think that […] could be improved there, or that's exactly what's happening in your project right now.’ If you have something specific in your hands, you can of course discuss whether one thing or another could be done better, yes? I can't talk to you about patient safety in general because that's not useful. No, of course safety should be given a very high priority. No mistakes should be made, no hospital germs, no infections, whatever else is involved. Patients should be informed about risks; we can talk about that for hours. But the more specific it becomes, the more specifically an advisory council can provide advice and perhaps even ensure that it gets through to patients.’* |
|  | INT#6: ‘*In the area of occupational safety and health, for example, there is an obligation to report accidents. And I don't know how it is with patient safety, whether there are things that are reported, must be reported and where, for example, there is an issue, is there a way, an obligation, that the patient advisory council also receives a transcript of all these incidents.’* |
|  | INT#1: ‘*Isn't it the case that patient safety would be explicitly on the agenda of the interaction? Well, we don't have one. Yes, well, patient safety is always there subliminally, in the things that concern us. But maybe it would be quite good if there were dedicated sessions on it [...] in practical terms.’* |
|  | INT#4: ‘*In terms of patient safety. In fact, we don't even have that as a topic for the advisory council right now. It's something that we haven't really looked at very intensively. [...]. I could certainly imagine inviting the colleague who is now in charge of the patient safety project here to the advisory council, just to present the project and to see what we need to look at more intensively in the area of oncology. Does everything run smoothly? Or have you had any experiences on the advisory council side where things haven't gone so well, or have you had any experiences? And where would you have to tighten up again, i.e. bring the topic to the advisory council in general?’* |
|  | INT#19: ‘*And then I think that would come, I think, in the next point, but it would be a format that we would also use to get the patient advisory councils in the mood for the topic of patient safety. [...] That something like this could perhaps be offered as a basic event in the patient advisory council meetings. (...) So that this knowledge, this awareness of this task that we definitely have, so that it's a practical thing where we can make a difference as patient representatives. (...) So that we are attuned to it.‘* |
|  | INT#18: ‘*But the patient advisory council also relies on concrete input from everyday life. And in the last ten years, I can think of two situations where we took a very concrete approach to processes that affected patient safety.’* |
|  | INT#17: ‘*We haven't discussed this topic in the patient advisory council yet, but I think that if I were to work in this area and be involved, I should know pretty well what patient safety is and where the focus should be. And as I said, I would like to take a closer look at it. It just hasn't been an issue until now.‘* |
| Roles and functions that PFACs could take on or assume | INT#3: ‘So, it is important that the advisory council finds itself in the development phase. A development phase where everyone can bring their strengths to the table. Where you look for similarities, where you formulate common goals, which you then present to the outside world. And you have to take this step alone, because you can't be under the control or supervision of the respective clinics, but you really have to do it alone. And then, when they have come to a conclusion, to coordinate this with the ideas of the board of directors or the board of directors of the respective clinic. Of course, the appropriate corrections have to be made.’’ |
|  | INT#13: ‘Structures are needed so that we are heard: ‘so that someone says, ‘This person is responsible for this, that person is responsible for that.’’ |
|  | INT#7: ‘*What we actually managed to do in the one advisory council, is to create a kind of pilot service and support, an orientation service for patients.’* |
|  | INT#1: ‘*And we represent the patient advisory council to the board of directors. Among other things, we are occasionally invited by the clinic, i.e. once a quarter, to board meetings, for example, and we always have the opportunity to bring concerns that arise from within or outside the patient advisory council to the board of directors.’* |
|  | INT#4: ‘*Then there are the areas of strategic direction, such as our cancer center, where the council is increasingly involved. There are certainly areas where the patient advisory council is not yet as involved, but the areas where it is consulted from time to time are growing.’* |
|  | INT 16: ‘*For example, we have now developed a flyer for location 1 in general on my initiative. This was very important to me. And with other people, we did a really, really good job, I think. And you could always do something like that for patient safety. You could somehow provide a framework for that*.‘ |
|  | INT 6: ‘*I come from an oncology background, as a patient, so to speak, and patient safety is very important in treatment, in terms of what medication, when, how, what chemotherapy, which? I can relate to that from my own experience. […]For me, this also includes the involvement of relatives, which also has to do with patient safety. And I think that we as patients can raise all these issues in the patient advisory council and make it clear to the clinic that patient safety is not just about hygiene or whether the medication is being given correctly, but that the whole context of the treatment plays an important role for us as patients.’* |
|  | INT#2: ‘*The first thing I would see in this position, and perhaps the most important, is collaboration in the area of quality management. Where do you get the requests for additional services? They come right to the table. I don't know how many requests come in on a weekly and monthly basis, what problems are identified, and what is done about them.’* |
|  | INT#9: ‘*So, yes, for example, in terms of discharge management or new patient admission management, we have been actively involved in the development of something that patients receive, such as a kind of introductory guide with contact persons and so on.’* |
|  | INT#6: ‘*This was about revising research, profiles, etc. and was immediately taken up and implemented.’* |
|  | INT#14: ‘*We also have a lot of non-German patients here in the city, and in that case, for example, we make sure that a native speaker is present to translate.’* |
|  | INT#14: ‘*Then I would say, first of all, that I think it is very important, if there is an opportunity and if the patient wants it, to be present at really important doctor's appointments. I think that's very important*.’ |
|  | INT#4: ‘*So, patients in the oncology area should be screened, regardless of whether a psycho-oncologist is consulted or not. [...] And here, too, we present the questionnaires and the approach to the patient advisory council, for example, how we now plan to increase the number of screenings. Are these questions easy to understand or do we need to phrase them differently? Or do we need to offer aftercare?‘* |

## **Table B. Qualitative quotes for influencing factors on PFACs’ work**

| **Theme** | **Illustrative Quote** |
| --- | --- |
| Recruitment and Personal Requirements of PFAC Stakeholders | INT#14: ‘‘*The problem is just that there are too few people [...] to do this volunteer work. And this has been the case for years, and at the moment there are nine of us on the patient advisory council. And that is just not enough for a big clinic like this.’* |
|  | INT#19: ‘‘*So you really have to take what each person can bring, what each person can do given their health and their limitations [...], because at some point they will not feel well and they will not be able to participate. And then, of course, it is also difficult to find patients who can really work seriously in the patient advisory council.*’ |
|  | INT#19: ‘‘*We need to get in touch with the organ centers to be more visible and to get patients who are able. At the moment, we take the patients we can get, so the patient advisory council is far from full, because there are simply not enough patients who want to participate*.‘ |
| Resources | INT#16: ‘‘*If these are really constructive suggestions, then they are also willing to implement them. It just shouldn't cost much. It shouldn't cause any particular difficulties within the clinic.’* |
|  | INT#12: ‘‘*That means that we get an allowance […] for attending meetings, for attending congresses, for attending council meetings [...]. I think that's good, because I think Germany is a country that relies a little bit on volunteer work - whether it's in associations or in politics. [...] Then we are grateful, and the wet handshake has to suffice. Uh, no, that's not enough […]. And it doesn't have to be rewarded in a way that makes us rich. For God's sake. But at some point I said: ‘'If I can't go to the shops and I can't cook because I'm stuck in some meetings, I'd like to have at least 10 Euros for a pizza I can order.'* |
|  | INT#18: ‘‘*There is always a lack of those resources and it is, frankly, illusory to think that with the current level of resources we can take it to the point where the patient perspective can really have a significant positive impact on patient safety. So [...] you would effectively have to pay people [...] a salary [...]. These are things that are just not feasible at the moment.*’ |
|  | INT#8: ‘‘*I don't know if there is [...] a regular office hour, where one or two of us are regularly available on a certain day in a certain room, where we can be approached and met. Yes, I would like that. Where patients can come to us and say, here, we have a problem and we need help.*’ |
|  | INT#7: ‘‘*[...] space is certainly [something that] needs to be organized, [...]. So I think that's a hurdle for the patient advisory council to start with, to say I have to see where I can get the space from.*’ |
|  | INT#6kY7u ‘*One thing is actually important: ‘that the members of the patient advisory council also have corresponding networks, fixed points of contact within the organizations who are the right people to talk to on the individual topics*.’ |
|  | INT#4: ‘‘*I think it would be really good if we could just have one for those who can't participate online and then have the option to really have the technology to be able to participate well in a hybrid way. That's something we need to work on a little bit to make it work properly*.‘ |
|  | INT#18: ‘‘*Actually, our next task is to organize a training course to further develop our self-help [...] but when should we do it? We don't have the resources [...]. Although there are [...] training opportunities [...] that already exist. So, the question is whether something else needs to be organized. We definitely support the formation of networks. Just through the structures that come before and after. That is the basic idea, to develop a network and to promote communication between the groups.*’ |
| System-related Conditions | INT#16: ‘‘*It is a step in the right direction that they are trying to involve patient representatives in projects and research. But it's also because otherwise you don't get funding. It's brutal. No money without our [patient] participation. So they include us.*’ |
|  | INT#2: ‘‘*But this [patient participation] is also a requirement for submitting an application [for research projects]. There is pressure there. And you probably don't have that in the clinic, in the care sector.*’ |
|  | INT#17: ‘‘*Well, you should at least be able to look at the process. (...) Yes, it is also important to see the patient's room again today, to see what it looks like and to be able to assess the standards, whether it is good or not so good. So that you simply have contact with it, which we don't have at all now.*’ |
|  | INT#19: ‘‘*[...] complaint management, that we actually have access there or that we are closely involved so that we know what the patient safety failures are.’*. |
|  | INT#19: ‘‘*But there are moments when we sense that there is also resistance. 'Now they want to say something about it too.' So, we really need to build the reputation of the patient advisory council and somehow manage to get people to say 'Hurray, they're getting involved!*’ |
|  | INT#2: ‘‘*[...] so I have the impression that we are not known either to the public or to patients and their families.*’ |
